# Structural basis for nucleosome binding and catalysis by the yeast Rpd3S/HDAC holoenzyme

**DOI:** 10.1038/s41422-023-00884-2

**Published:** 2023-10-16

**Authors:** Yueyue Zhang, Mengxue Xu, Po Wang, Jiahui Zhou, Guangxian Wang, Shuailong Han, Gang Cai, Xuejuan Wang

**Affiliations:** 1 The First Affiliated Hospital of USTC, MOE Key Laboratory for Cellular Dynamics, Division of Life Sciences and Medicine, University of Science and Technology of China, Hefei, Anhui China; 2 Key Laboratory of Anhui Province for Emerging and Reemerging Infectious Diseases, Hefei, Anhui China

**Keywords:** Cryoelectron microscopy, Histone post-translational modifications

Dear Editor,

Histone deacetylases (HDACs) are evolutionally conserved enzymes that remove acetyl modifications from histones and play a central role in epigenetic gene silencing.^1^ Class I HDACs are promising targets for epigenetic therapies for a range of diseases such as cancers, inflammations, infections, and neurological diseases.^2^ Yeast Rpd3 is the founding member of class I HDACs, which forms two distinct complexes: the ∼1.2 MDa Rpd3L deacetylating histones at promoter regions, and the ∼0.6 MDa Rpd3S targeting transcribed regions to suppress intragenic transcription initiation.^3,4^ Rpd3S consists of three core proteins: Rpd3, Sin3, and Ume1^5^ along with two dedicated chromatin binding subunits: Eaf3 and Rco1.^6^ The structures of the yeast Rpd3S complex and its human homolog Sin3B complex have been recently reported.^7,8^ Here, we report the cryo-electron microscopy (cryo-EM) structure of the Rpd3S holoenzyme binding a nucleosome at 3.7 Å resolution (Supplementary information, Table S1). The structure illuminates an intact H3 tail (1–24 aa) binding to the Rpd3S complex in a catalysis-competent conformation, with the H3K18 residue poised for catalysis.

The endogenous Rpd3S holoenzyme was affinity-purified to high homogeneity (Supplementary information, Fig. S1). A mononucleosome containing methyl-lysine analogs (H3Kc36me3) with a 70-bp DNA link at one end was assembled. The Rpd3S incubated with an excess nucleosome was fractionalized by gel filtration and then concentrated (Supplementary information, Fig. S2). The resulting Rpd3S–Nucleosome (Rpd3S–Nuc) without any cross-linking interference was directly subjected to cryo-EM analysis (Supplementary information, Fig. S4). A cryo-EM reconstruction to an average resolution of 3.7 Å was obtained, which enabled de novo model building of the Rpd3S–Nuc complex (Fig. 1b; Supplementary information, Figs. S4 and S5).

**Fig. 1:**
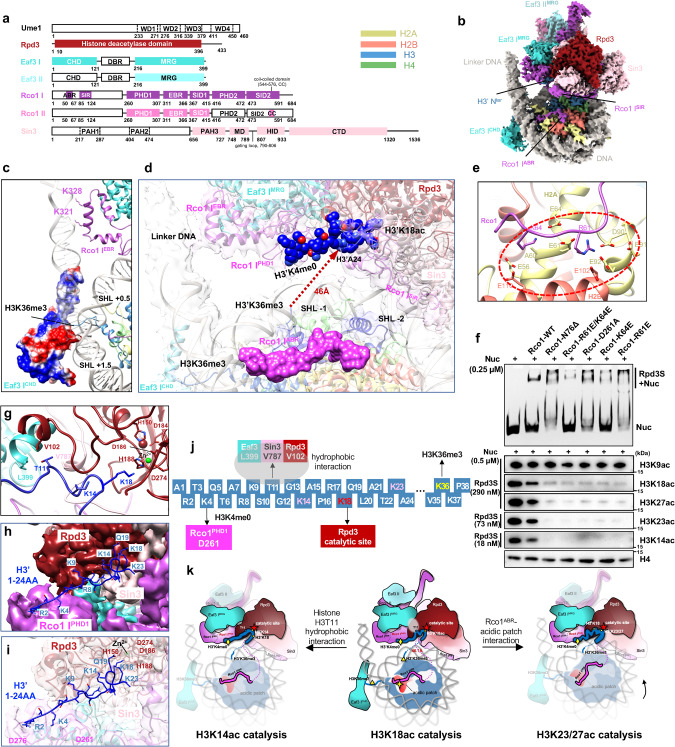
Nucleosome binding and catalysis by the yeast Rpd3S/HDAC holoenzyme. **a** Schematics showing domains and functional regions of Rpd3S subunits. Proteins are color-coded, with those not structurally resolved in white. WD, tryptophan-aspartic acid; DBR, DNA binding region. The Eaf3–Rco1 copy II is in less saturated color than the Eaf3–Rco1 copy I. The color scheme for Rpd3S subunits and histones is used throughout all figures. **b** Front view of the cryo-EM composite map of the Rpd3S–Nuc complex. Mononucleosomes containing methyl-lysine analogs (H3Kc36me3) with 70-bp DNA link at one end are used in the assembly. **c** Close-up view of interactions between Eaf3 I^CHD^ with H3K36 tail and nucleosomal DNA and Rco1 I^EBR^ with extranucleosomal DNA. The electrostatic surface potential (–/+3.000) is shown on Eaf3 I^CHD^. The positively charged surface of the Eaf3 I^CHD^ packs against the nucleosome DNA between SHL +0.5 and SHL +1.5. Several lysine residues of Rco1 I^EBR^ bind the extranucleosomal DNA. **d** Multivalent recognition of nucleosomal linker DNAs, histone acidic patch, and H3’K4me0 by Rco1 subunit. The Rco1 I^ABR^ interacting with the nucleosome acidic patch is depicted on the surface. The H3’ substrate (1–24 aa) is highlighted in ball-and-stick model. The linear distance of the invisible H3 tail (25–37 aa) is illustrated. **e** Close view showing that the Rco1 I^ABR^ contacts the acidic patch of the nucleosome (dotted red circle). **f** EMSA of the nucleosome binding activity of WT Rco1 and its mutants (top panel). 0.25 μM H3K36me3-modified nucleosome and equal amounts of Rpd3S complex were used. Western blot analysis of HDAC activity of WT Rpd3S and its mutants on H3K36me3 and H3/H4 hyperacetylated nucleosome (bottom panel). The concentration of nucleosome and the respective Rpd3S is indicated on the left. **g** Close-up view of the H3 tail penetrating into the active site of the Rpd3S complex. The H3T11 lies in a hydrophobic pocket corresponding to the interface among Eaf3(Leu399), Sin3(Val787), and Rpd3(Val102). The catalytic Zn^2+^ and its stabilizing residues of Rpd3 are highlighted. **h**, **i** A detailed view of interactions between an intact H3’ tail (1–24 aa) and the Rpd3S complex. The Rpd3S complex is shown in ribbon and transparency surface (**h**) and solid surface representation (**i**), respectively. **j** The diagram of the observed interaction between Rpd3S and the H3’ substrate. **k** A cartoon depicting the mechanism underlying how Rpd3S selects and catalyzes the specific lysine residues of H3 tails. The middle panel corresponds to the directly visualized H3’K18 catalytic state. The left and right panels correspond to the putative H3’K14ac and H3’K23/27ac catalytic states, respectively.

Notably, the structure of the Rpd3S complex discloses two copies of Rco1 and two copies of Eaf3 (Fig. 1a, b). Both Rco1subunits are required for full functionality of Rpd3S.^9^ The two copies of Rco1–Eaf3 dimerize through the Rco1 C-terminal coiled-coil domain (CC) and the dimer interface packs against Sin3 paired amphipathic α-helix 3 (PAH3) (Supplementary information, Figs. S6a, d, e and S7). Sin3 is the scaffold subunit, which wraps around the catalytic Rpd3/HDAC via its HDAC interacting domain (HID), middle-domain (MD), and C-terminal domain (CTD) (Supplementary information, Fig. S6a, c, e). Ume1 only peripherally contacts Sin3 and is only visible at a low threshold (Supplementary information, Figs. S3 and S8).

The structure clearly resolves several Rco1 regions interacting extensively with other subunits and nucleosome substrates, especially several uncharacterized domains. Rco1 contains nucleosome acidic patch binding region (ABR, 50–67 aa), Sin3 interacting region (SIR, 85–124 aa), PHD1(plant homeodomain 1, 260–307 aa) binding to H3K4me0 tail, Eaf3 binding region (EBR, 311–366 aa), Sin3 interacting domain 1 (SID1, 367–415 aa) which interacts with Sin3 gating loop, PHD2 (416–472 aa) binding to Eaf3 MRG (MORF4 related gene) and Rpd3, and SID2 (473–591 aa) responsible for Rco1–Rco1 dimer formation (Fig. 1a; Supplementary information, Figs. S6a, b, e and S13c). Rco1 interacts with Eaf3 and also packs against Sin3–Rpd3 interface, implicating a putative allosteric regulation of Rco1 on Rpd3/HDAC activity.

There are four major Rpd3S–Nuc interfaces. Sensing extranucleosomal DNA is achieved by Eaf3 chromatin binding domain (CHD) and Rco1 EBR (Fig. 1c). Eaf3 CHD extends to and separates from the Eaf3 MRG. Besides H3K36me2/3, Eaf3 CHD makes extensive interaction with the wrapped DNA at SHL (+0.5–+1.5) (Fig. 1c; Supplementary information, Fig. S9). In addition, Rco1 residues K321 and K328 (within EBR) also directly bind the backbone of extranucleosomal DNA (Fig. 1c). Recognition of nucleosomal DNA is mainly achieved by Sin3 (Fig. 1d; Supplementary information, Fig. S10). Sin3 residues K936, K940, K941, and K1244 within CTD bind the DNA backbone at SHL –2 (Supplementary information, Fig. S10b). Notably, H3 tails generally extend through the DNA gyres at SHL –2 and SHL +0.5/+1.5.^10^ During Rpd3S engagement, these two DNA contact points with H3 tails are bound by Sin3 CTD and Eaf3 CHD. Meanwhile, the Rpd3/HDAC active site is locked by Sin3 into a fixed position pointing to the SHL –1, facilitating the released H3 tail extending into the catalytic site (Fig. 1d; Supplementary information, Fig. S15).

The function of the Rco1 N-terminal is largely uncharacterized, which contains an intrinsically disordered fragment. Our newly defined Rco1 ABR (50–67 aa) extends from the Rpd3S core and anchors onto the surface of the acidic patch through Rco1 R61 and K64 residues (Fig. 1b, d, e; Supplementary information, Fig. S11b). The nucleosome acidic patch is important for binding many chromatin-associated factors.^11^ Surprisingly, the N-terminal of ABR, through side chains of R50 and R51, binds the backbone of wrapped DNA at SHL –6.5 (Supplementary information, Fig. S12). Therefore, ABR is involved not only in the recognition of histone acidic patch but also in interaction with nucleosomal DNA. Except for ABR, part of the flexible region connecting Rco1 PHD1 and ABR is also well-defined in our structure, named as SIR (85–124 aa), which lies along the surface of Sin3 HID (Fig. 1b; Supplementary information, Fig. S11a).

Our structure clearly resolves an intact H3 tail (1–24 aa) binding to the Rpd3S, with the unexpected H3K18 residue poised for catalysis (Fig. 1d, h, i; Supplementary information, Fig. S13). The ϵ-amino group of the H3K4 side chain directly interacts with the carboxyl group of the D261 of Rco1 PHD finger (Fig. 1d, h, i; Supplementary information, Fig. S13b), which recognizes unmodified H3K4.^12^ Methylation of the H3K4 residue shall destabilize the electrostatic interaction. Therefore, only the H3K4me0 tail could be grabbed by Rco1, which explains why Rpd3S is restricted from binding to promoter nucleosomes marked with H3K4me2/3. In addition, H3Q5 also interacts with Rpd3 K98 residue (Supplementary information, Fig. S13b). Through recognition of H3K4me0 by Rco1 and H3Q5 by Rpd3, a histone H3 tail is delivered into the Rpd3/HDAC active site for catalysis. Since H3K4-Q5 is immobilized, H3K9 could not access the deep Rpd3 catalytic pocket, which is consistent with the absence of deacetylation on H3K9ac^13^ (Fig. 1f, bottom panel; Supplementary information, Fig. S17).

The H3 tail (1–24 aa) enters the Rpd3S active site and ties a small knot around H3T11 (Fig. 1g). A cluster of hydrophobic residues from Eaf3 (Leu399), Sin3 (Val787), and Rpd3 (Val102) accommodate H3T11. Since the H3T11 is immobilized by the hydrophobic pocket formed by Eaf3, Sin3, and Rpd3, the H3K14 cannot access the Rpd3S catalytic center in this conformational state. Our structure discloses that only H3K18 residue is observed to access Rpd3 catalytic residue for catalysis. The lysine side chain of H3K18 points toward the catalytic Zn^2+^ stabilized by H188, D186, and D274 of Rpd3 (Fig. 1g, i; Supplementary information, Figs. S13c, d and S14). Consistently, the in vitro enzymatic assay suggested that H3K18ac can be catalyzed by Rpd3S (Fig. 1f, bottom panel; Supplementary information, Fig. S17) and in vivo Rpd3S is critically responsible for maintaining H3K18ac level.^13^ The distance between H3A24 to H3P38 is 46 Å (Fig. 1d; Supplementary information, Fig. S15). The distance restraint of the dynamic H3 tail (25–37 aa) could only allow the invisible 13 residues in a fully extended conformation. Therefore, in this binding mode, due to distance limitations, the H3K23 and H3K27 extended from SHL –1 cannot approach the Rpd3 catalytic pocket either.

The H3K18Ac is a marker of cancer progression and potential target of anti-cancer therapy.^14^ Enzymatic assays revealed that the deacetylation activities of wild-type (WT) Rpd3S on H3K18ac and H3K27ac are comparably low. Whereas, its activity on H3K14ac and H3K23ac is significantly higher, and noticeably less Rpd3S can achieve efficient deacetylation (Fig. 1f, bottom panel; Supplementary information, Fig. S17). The structural observation of H3K18 accessing the WT Rpd3S catalytic center probably represents the most stable binding state of the Rpd3S–Nuc assembly, which corresponds to a crisp sharp band in the electrophoretic mobility shift assay (EMSA) (Fig. 1f, upper panel). The stable binding state of H3K18 in the Rpd3S active site leads to low catalytic activity. The two closest sites to H3K18 are H3K14 and H3K23, which only require minimal conformational changes to directly contact the Rpd3S active center. Therefore, these two sites have the highest catalytic activity. H3K27, which is further away from K18, requires much more conformational changes, resulting in lower activity.

The visualized H3K18 catalytic state is achieved mainly by Rco1. The Rco1 mutants of ABR and of PHD1 obscured the gel-shift bands of the Rpd3S–Nuc assembly and the smear bands indicated a heterogeneous population of Rpd3S–Nuc binding states. Therefore, these Rco1 mutants altered their specificity toward K36-methylated nucleosomes. Compared to WT Rco1, the Rco1-N76Δ (N-terminal 1–76 aa deletion), Rco1-D261A, and Rco1-R61E increased affinity to K36-methylated nucleosomes, whereas Rco1-R61E/K64E and Rco1-K64E marginally reduced affinity to nucleosomes (Fig. 1f, upper panel; Supplementary information, Fig. S16). The in vitro enzymatic analysis using the nucleosomal substrates harboring combinatorial multiple acetylations on both H3 and H4, suggested that the catalytic activity of Rco1 mutants towards histone H3 are largely boosted (Fig. 1f, bottom panel; Supplementary information, Fig. S17). Whereas, nucleosomes devoid of any acetylation modifications were utilized in the EMSA. The discrepancy of substrate affinity and deacetylation activity of Rco1 mutants may result from the distinct nucleosomal substrates and the variation of the incubation conditions. Nevertheless, our functional study of Rco1 mutants corroborates our structural observations that Rco1 indeed plays a critical and central role in nucleosome binding and deacetylation catalysis.

During the submission of this manuscript, Guan et al. published two 4.0 Å structures of the Rpd3S–Nuc complex^13^ and Dong et al. reported several structures of Rpd3S–Nuc with different DNA linkers.^15^ The Eaf3 II CHD bound to nucleosome is observed in these reported structures, which is absent in our structure (Supplementary information, Fig. S18). However, these models could not provide information on the Rco1’s interface with the histone core nor the histone substrate immobilized in the Rpd3S’s substrate binding groove. An intact H3 tail (1–24 aa), the Eaf3 II MRG, Rco1 II PHD1-SID1, Rco1 I SID1 (379–399 aa), and Rco1 I ABR (50–67 aa) can only be visualized in our structure. The reason behind the structural differences could be the variation in the nucleosome substrate and different assembly conditions.

Besides H3K18ac, Rpd3S can also catalyze deacetylation of H3K14ac, H3K23ac, and H3K27ac (Fig. 1f, j). The architecture of the yeast Rpd3S bound to the nucleosome substrate and putative allosteric conformational changes could rationalize catalytic promiscuity of Rpd3S/HDAC holoenzyme. The cooperative conformational changes of Eaf3, Rpd3, and Sin3 may destabilize the hydrophobic pocket that holds H3T11 and result in straightening the originally bent H3 tail. These allosteric conformational changes could further enable H3K14ac to enter the active site for catalysis (Fig. 1k, left panel). The intrinsically disordered fragment (68–84 aa) connecting Rco1 ABR with Rco1 SIR and the anchored nucleosome acidic patch by Rco1 ABR may facilitate H3 K23/27ac to move towards the catalytic Rpd3S residues (Fig. 1k, right panel). Our study clarifies the architecture of the Rpd3S–Nuc complex, illuminating multivalent nucleosome engagement and allosteric conformational changes underlining the substrate specificity and catalytic promiscuity of Rpd3S/HDAC holoenzyme. Notably, Rco1 plays a critical role in nucleosome binding and site-specific catalysis. We propose that Rco1 may function as a hot spot for novel Rpd3S/Sin3B inhibitor development.

### Supplementary information

SUPPLEMENTAL MATERIAL

## Data Availability

Coordinates and EM maps are deposited in the Protein Data Bank under accession codes EMD-35456 (Rpd3S apo state 1), EMD-35457 (Rpd3S apo state 2), EMD-35458 (Rpd3S apo state 3), EMD-35449, PDB ID 8IHM (Eaf3 CHD bound to the nucleosome), EMD-35450, PDB ID 8IHN (Rpd3S core complex), EMD-35455, and PDB ID 8IHT (Rpd3S bound to the nucleosome).
