# Supplementary material for: Structural basis for nucleosome binding and catalysis by the yeast Rpd3S/HDAC holoenzyme
Source: Cell Res. 2023 Oct 16;33(12):971–4. doi: 10.1038/s41422-023-00884-2 (PMC10709394; doi:10.1038/s41422-023-00884-2)
Supplement: Supplementary file 1 — SUPPLEMENTAL MATERIAL [file 41422_2023_884_MOESM1_ESM.pdf]

Supplementary Information

Structural basis for nucleosome binding and catalysis by the yeast Rpd3S/HDAC holoenzyme

Yueyue Zhang[1†], Mengxue Xu[1†], Po Wang[1], Jiahui Zhou[1], Guangxian Wang[1], Shuailong Han[1], Gang Cai[1,2*] and Xuejuan Wang[1,2*]

[1] The First Affiliated Hospital of USTC, MOE Key Laboratory for Cellular Dynamics, Division of Life Sciences and Medicine, University of Science and Technology of China, Hefei, Anhui, 230001, China;

[2] Key Laboratory of Anhui Province for Emerging and Reemerging Infectious Diseases, Hefei, 230027, China.

† These authors contributed equally to this work.

* To whom correspondence may be addressed. Email: xuejuan@ustc.edu.cn and gcai@ustc.edu.cn

Purification of endogenous Rpd3S complex

The Rpd3S complex was purified from a yeast strain containing a 2xFlag tag at the C terminus of Rco1 subunit. The tagged yeast strain was grown in 10 L YPD medium overnight at 30°C. Cells were collected by centrifugation and resuspended in lysis buffer (50 mM Tris pH 7.6, 300 mM ammonium sulfate, 1 mM EDTA, 10 uM $ZnCl_2$, 10% glycerol and protease inhibitor cocktail). The cell suspension was frozen in liquid nitrogen and milled to powder using a cryogenic grinder (Spex sample prep 6875). The powder was thawed at 4°C and centrifuged at 16,000 g for 30 min to remove cell debris. The supernatant was centrifuged in a Beckman Optima XE-90 ultracentrifuge with a Beckman Type 45 Ti rotor at 40,000 rpm for 2 hours at 4°C. The clarified supernatant was selectively precipitated in 30%-55% ammonium sulfate and resuspended in binding buffer (25 mM Tris pH 7.6, 100 mM ammonium sulfate, 1 mM EDTA, 10 uM $ZnCl_2$, 10% glycerol and protease inhibitor cocktail). For the affinity purification, 0.5 ml Anti-FLAG M2 affinity beads (Sigma) were incubated with the supernatant at 4°C for 2 hours. The beads were next washed and resuspended in 1ml elution buffer (25 mM HEPES pH 7.6, 100 mM ammonium sulfate, 1 mM EDTA, 10% glycerol). Protein elution using 10 mM 3xFLAG peptide (Sigma) was performed at 4°C for 3 hours with gentle shaking. The eluate was concentrated and loaded onto a Superose 6 10/300 GL column (GE Healthcare Life Sciences) for further purification with SEC buffer (25 mM HEPES pH 7.6, 100 mM NaCl, 1 mM EDTA, 10% glycerol). The peak fractions containing the Rpd3S complex were combined and aliquoted, flash frozen in liquid nitrogen and stored at -80°C. For each purification step, the Rpd3S complex was monitored with purity and structural integrity by SDS-PAGE and EM examination.

Preparation of histone H3 modified with Kc36me3

To generate the histone H3 with Kc36 trimethylation, mutations (K36C, C110A) were introduced into the H3 sequence by site-directed mutagenesis. The cysteine was alkylated to create *N*-methylated aminoethylcysteine residues, an analog of methyl-lysine, as previously described [1]. Briefly, 10 mg freeze-dried histones were dissolved in alkylation buffer (1 M HEPES pH 7.8, 4 M chloride, 10 mM D/L-methionine) and reduced with DTT at 37°C for 1 hour. 100 mg (2-bromoethyl) trimethylammonium bromide (Sigma) was added and incubated at 50°C for 5 hours with occasional mixing. The reactions were

quenched with 5 mM β-mercaptoethanol and dialyzed to 3 mM β-mercaptoethanol/H2O. The modified histone H3 was aliquoted, lyophilized, and stored at -80°C. The appropriate trimethylation was confirmed by western blotting using H3K36me3 antibody (Abcam: ab9050).

Preparation of mononucleosome with 216 bp DNA

Xenopus laevis histones H2A, H2B, H3-K36C/C100A and H4 were expressed respectively in *E. coli* BL21(DE3) as previously described [2,3] with modifications. The histones were purified from the inclusion body using SP sepharose resin under denaturing condition, further purified using a YMC-Pack PROTEIN-RP column with HPLC (Waters: 2535q) and lyophilized. To prepare the octamer, histones H2A, H2B, H3-K36C/C100A and H4 were mixed in an equimolar ratio and dialyzed against the refolding buffer (10 mM Tris pH 7.5, 2 M NaC1, 1 mM EDTA, 5 mM β-mercaptoethanol). The octamer was purified by loading the mixture onto a Superdex 200 increase 10/300 GL column (GE Healthcare Life Sciences) with refolding buffer.

The 216 bp DNA fragment containing a linker DNA and a nucleosome positioning sequence (Widom 601) [4] was generated by PCR. The PCR product was further purified by ion-exchange chromatography (Mono Q 5/50 GL, GE Healthcare Life Sciences) followed by isopropanol precipitation. The precipitated DNA pellet was dissolved in water, aliquoted, and stored at -20°C.

The sequence of 216 bp DNA with Widom 601 DNA underlined is as follows:

5’-
ACCATCGTCAGTATTGACTGCAGACCTGAAGCTTGATATCGAATTCGCGTGTC
GCCCTTACTGGCCGCCCTGGAGAATCCCGGTGCCGAGGCCGCTCAATTGGTC
GTAGACAGCTCTAGCACCGCTTAAACGCACGTACGCGCTGTCCCCCGCGTTTT
AACCGCCAAGGGGATTACTCCCTAGTCTCCAGGCACGTGTCAGATATATACA
TCCTGT-3’

Mononucleosome reconstitution was performed according to previously published protocols [5] by salt-dialysis method, and purified by non-denaturing electrophoresis using a Prep Cell apparatus (Bio-Rad). The resulting nucleosome with H3Kc36me3 was concentrated to approximately 1 mg/ml and stored on ice until used. The histone octamers for the HDAC assay containing H3K36me3, H3K9acK14acK18acK23K27ac

and H4K5acK8acK12acK16ac modifications were a kind gift from Prof. Haitao Li.

Electrophoretic mobility shift assay

0.25 μM of H3K36me3 modified nucleosome and equal amounts of each form purified Rpd3S were incubated in EMSA buffer (20 mM HEPES-Na pH 7.5, 50 mM NaCl, 1 mM DTT) for 1 hour on ice. The Rpd3S-nucleosome complex was detected by electrophoresis on a 6% Native-PAGE gel in 0.2× TBE buffer at a constant voltage of 100 V for 1 hour. The gel was incubated for 10 mins in TS-GelRed (Tsingke Biotechnology) containing NaCl buffer and visualized using an Imaging System (Tanon-2500). The unbound nucleosome bands intensities were quantified using the ImageJ software. The mean values SD from three independent experiments were plotted.

HDAC assays

We measure the histone deacetylation activity of Rpd3S complex as previously described [6], in a buffer containing 20 mM HEPES pH 7.5, 150 mM NaCl, 0.2 mg ml^{-1} BSA, and 0.15 μg μl^{-1} salmon sperm DNA. Reactions were initiated by mixing Rpd3S complex at different concentrations with 0.5 μM hyperacetylated nucleosome (H3Kac, H4Kac and H3K36me3) and kept at 30 °C for 25 min. The reactions were stopped by the addition of SDS–PAGE loading buffer, then the samples were boiled for 5 min at 95 °C. Proteins were separated on 4–20% SDS-PAGE gel and transferred onto PVDF membrane. H3K9ac, H3K14ac, H3K18ac, H3K23ac, H3K27ac and H4 were detected by western blot with specific primary antibodies on separate gels. The signal on blots was developed with the western blotting substrate (Tanon, High-sig ECL).

Primary antibodies used: rabbit monoclonal anti-H3K9ac (Cell Signaling Technologies, 9649S, dilution: 1:1,000), rabbit monoclonal anti-H3K14ac (Cell Signaling Technologies, 7627T, dilution: 1:1,000), rabbit monoclonal anti-H3K18ac (Cell Signaling Technologies, 13998S, dilution: 1:1,000), rabbit polyclonal anti-H3K23ac (PTM BIO, PTM-115, dilution: 1:1,500), rabbit monoclonal anti-H3K27ac (Cell Signaling Technologies, 8173S, dilution: 1:1,000), mouse monoclonal anti-H4 (PTM BIO, PTM-1009, dilution: 1:1,000).

Secondary antibodies used: Goat Anti-Mouse IgG-HRP (Abmart, M21001L, dilution: 1:10,000), Goat Anti-Rabbit IgG-HRP (Abmart, M21002S, dilution: 1:10,000).

Cryo-EM sample preparation of Rpd3S-nucleosome complex

The Rpd3S was incubated with excess H3Kc36me3-modified nucleosome for 1 hour on ice. Excess nucleosome was removed by a Superose 6 10/300 GL column equilibrated in EM buffer (25 mM HEPES pH 7.6, 100 mM NaCl, 1 mM EDTA, 1 mM DTT, 2% glycerol). The fractions corresponding to Rpd3S-nucleosome complex were assessed by 6% Native-PAGE and SDS-PAGE, then collected and concentrated for cryo-EM grid preparation.

Cryo-EM grid preparation and data collection

For the Rpd3S complex, purified protein was diluted to approximately 50 μg/ml with the buffer (25 mM HEPES pH 7.6, 100 mM NaCl, 1 mM EDTA, 10% glycerol) and 3 μl of aliquots were applied onto freshly glow-discharged lacey carbon grids coated with a second layer of 3nm thick carbon film (TED PELLA). Samples were incubated on grids for 20 s, blotted for 3.5 s and plunged frozen in liquid ethane. Cryo-EM grids were prepared with an EMGP2 (Leica company) set to 8°C and 95% humidity. The data was acquired on a Titan Krios electron microscope (FEI) at 300 keV equipped with a K2 summit direct electron detector (Gatan). A total of 4,724 movies were automatically recorded using SerialEM software at a nominal magnification of 18,000x, yielding a pixel size of 1.35 Å in counting mode, with defocus values ranging from -2.5 to -3.5 μm. The data was recorded with a total dose of 59 electrons per Å^2, recording 36 frames.

For the Rpd3S-nucleosome complex, aliquots of 3 μl concentrated sample were loaded onto glow-discharged Quantifoil R2/1 Au 400 grids. After incubation for 20 s, the grids were blotted for 4 s and plunged frozen in liquid ethane. Grids were transferred to a Titan Krios transmission electron microscope (Thermo Fisher) operating at 300 kV equipped with a Gatan Gif Quantum energy filter. Cryo-EM data was collected with EPU software through a Gatan K3 direct electron detector in counting mode at 81,000 x nominal magnification, resulting in a physical pixel size corresponding to 1.07 Å, with a defocus range of -1.8 to -2.8 μm. Exposure of 3.8 s was dose-fractionated into 32 moves frames, leading to a total accumulated dose of 53 electrons per Å^2 on the specimen. A total of 2,818 movies were saved as unprocessed Tiff files for analysis.

Image processing

The Rpd3S dataset：

Individual movie frames were aligned using MotionCor2 [7]. CTF parameters were estimated using CtfFind4 [8]. Particles were picked in Gautomatch (by K.Z., under development) using 2D averages obtained from negative staining data. Subsequent image processing was carried out in RELION-4.0-beta-2 [9] and cryoSPARC v3.3.2 [10]. A total of 876,212 particles were extracted by four-times downscaling resulting in the pixel size of 5.4 with a box size of 84 x 84 pixels. Multiple rounds ab-initio reconstruction and heterogeneous refinement were run to get the best particles. The unbinned 73,857 particles were re-extracted and divided into three classes using heterogeneous refinement in cryoSPARC. Then the particles (24,648 particles; 25,950 particles; 23,259 particles) of these three conformations were reconstructed using non-uniform refinement, respectively. The final models were refined to an overall resolution of 9.84 Å, 8.68 Å and 8.82 Å.

The Rpd3S-nucleosome dataset：

The movies were imported into RELION-4.0-beta-2, and the whole-frame motion correction was carried out by the RELION's own implementation with dose weighting. Contrast transfer function (CTF) parameters were estimated using CTFFIND4. Subsequently, micrographs were rejected with extreme defocus values, too much contamination or aggregation. Two particle subsets were initially picked using the Gautomatch with the templates of Rpd3S and nucleosome, respectively, from the remaining good 2,635 micrographs. The two subsets were extracted by four-times downscaling resulting in the pixel size of 4.28 with a box size of 84 x 84 pixels, and combined. The particles within the minimum inter-particle distance 30 Å were removed leaving only one. Multiple rounds of reference-free 2D classification were run to remove bad particles, and the following processes were performed using cryoSPARC v3.3.2. After Ab-Initio reconstruction and heterogeneous refinement, a set of 130,804 particles was re-extracted with the pixel size of 1.07. Further 3D classification was performed to get homogeneous nucleosome-bound particles. The model was refined to an overall resolution of 4.06 Å with 68,446 particles. To further improve the final resolution, a series of 2D projections of the 4.06 Å map were used as a template to re-pick particles

using Gautomatch. Subsequent processes were similarly performed as described above. A final set of 107,252 Rpd3S-nucleosome complex particles was selected to perform a final 3D refinement, leading to a map with resolution at 3.72 Å. The same particles were further used for particle subtraction, the Rpd3S core complex and nucleosome-Eaf3 complex, and further local refinement was carried out to obtain 3.37 Å and 3.58 Å maps. The resolution values reported for all reconstructions were based on the gold-standard Fourier shell correlation (FSC) 0.143 criterion.

Model building and refinement

A nucleosome structure (PDB 6S01) [11] and the available structures of Rpd3S from AlphaFold protein structure database [12,13] were fitted into the refined 3D reconstruction map using UCSF Chimera [14] and then manually rebuilt in Coot [15]. The remainder of the molecules, including Rco1 N-ter, Rpd3 C-ter and linker DNA, were manually built based on high-resolution maps in Coot. The model was subject to real-space refinement in Phenix 1.18 [16]. Statistics of the map reconstruction and model refinement were shown in the Supplementary Information Table. Structural analysis was performed in Coot, and figures were prepared using UCSF Chimera and ChimeraX [17].

Yeast genetics

To generate the Rco1 mutant strains (R61E,K64E,R61E/K64E,D261A) and Rco1-N76 Δ strain (1-76aa deletion), the full-length of Rco1 sequence was cloned into the vector pFA6a-TRP1. The homology recombination templates containing a 2xFlag tag at the C terminus of Rco1 were amplified independently by PCR from the modified plasmid. The PCR products were transformed into *Saccharomyces cerevisiae* chemically competent cell W303a (MATa ade2-1 can1-100 his3-11 leu2-3 leu2-112 trp1-1 ura3-1) by the lithium acetate method and yeast cells were selected using Trp minus medium. All deletant and mutants were confirmed by PCR and DNA sequencing.

All modified strains were used for Rpd3S complex endogenous purification, as described above.

Supplementary Reference:

1. Simon, M. D. *et al.* The site-specific installation of methyl-lysine analogs into recombinant histones. *Cell* **128**, 1003-1012 (2007). https://doi.org:10.1016/j.cell.2006.12.041
2. Zhou, B. R. *et al.* Atomic resolution cryo-EM structure of a native-like CENP-A nucleosome aided by an antibody fragment. *Nat Commun* **10**, 2301 (2019). https://doi.org:10.1038/s41467-019-10247-4
3. Dyer, P. N. *et al.* Reconstitution of nucleosome core particles from recombinant histones and DNA. *Methods Enzymol* **375**, 23-44 (2004). https://doi.org:10.1016/s0076-6879(03)75002-2
4. Lowary, P. T. & Widom, J. New DNA sequence rules for high affinity binding to histone octamer and sequence-directed nucleosome positioning. *Journal of Molecular Biology* **276**, 19-42 (1998). https://doi.org:DOI 10.1006/jmbi.1997.1494
5. Luger, K., Rechsteiner, T. J. & Richmond, T. J. Preparation of nucleosome core particle from recombinant histones. *Methods Enzymol* **304**, 3-19 (1999). https://doi.org:10.1016/s0076-6879(99)04003-3
6. Guan, H. *et al.* Diverse modes of H3K36me3-guided nucleosomal deacetylation by Rpd3S. *Nature* (2023). https://doi.org:10.1038/s41586-023-06349-1
7. Zheng, S. Q. *et al.* MotionCor2: anisotropic correction of beam-induced motion for improved cryo-electron microscopy. *Nat Methods* **14**, 331-332 (2017). https://doi.org:10.1038/nmeth.4193
8. Rohou, A. & Grigorieff, N. CTFFIND4: Fast and accurate defocus estimation from electron micrographs. *J Struct Biol* **192**, 216-221 (2015). https://doi.org:10.1016/j.jsb.2015.08.008
9. Scheres, S. H. RELION: implementation of a Bayesian approach to cryo-EM structure determination. *Journal of structural biology* **180**, 519-530 (2012). https://doi.org:10.1016/j.jsb.2012.09.006
10. Punjani, A., Rubinstein, J. L., Fleet, D. J. & Brubaker, M. A. cryoSPARC: algorithms for rapid unsupervised cryo-EM structure determination. *Nat Methods* **14**, 290-296 (2017). https://doi.org:10.1038/nmeth.4169
11. Wang, H. B., Farnung, L., Dienemann, C. & Cramer, P. Structure of H3K36-methylated nucleosome-PWWP complex reveals multivalent cross-gyre binding. *Nat Struct Mol Biol* **27**, 8-+ (2020). https://doi.org:10.1038/s41594-019-0345-4
12. Jumper, J. *et al.* Highly accurate protein structure prediction with AlphaFold. *Nature* **596**, 583-+ (2021). https://doi.org:10.1038/s41586-021-03819-2
13. Varadi, M. *et al.* AlphaFold Protein Structure Database: massively expanding the structural coverage of protein-sequence space with high-accuracy models. *Nucleic Acids Research* **50**, D439-D444 (2022). https://doi.org:10.1093/nar/gkab1061
14. Pettersen, E. F. *et al.* UCSF Chimera--a visualization system for exploratory research and analysis. *J Comput Chem* **25**, 1605-1612 (2004). https://doi.org:10.1002/jcc.20084
15. Emsley, P. & Cowtan, K. Coot: model-building tools for molecular graphics. *Acta Crystallogr D Biol Crystallogr* **60**, 2126-2132 (2004). https://doi.org:10.1107/S0907444904019158
16. Adams, P. D. *et al.* PHENIX: a comprehensive Python-based system for macromolecular structure solution. *Acta Crystallogr D Biol Crystallogr* **66**, 213-221 (2010). https://doi.org:10.1107/S0907444909052925

17 Goddard, T. D. *et al.* UCSF ChimeraX: Meeting modern challenges in visualization and analysis. *Protein Sci* **27**, 14-25 (2018). https://doi.org:10.1002/pro.3235

Supplementary information, Fig. S1

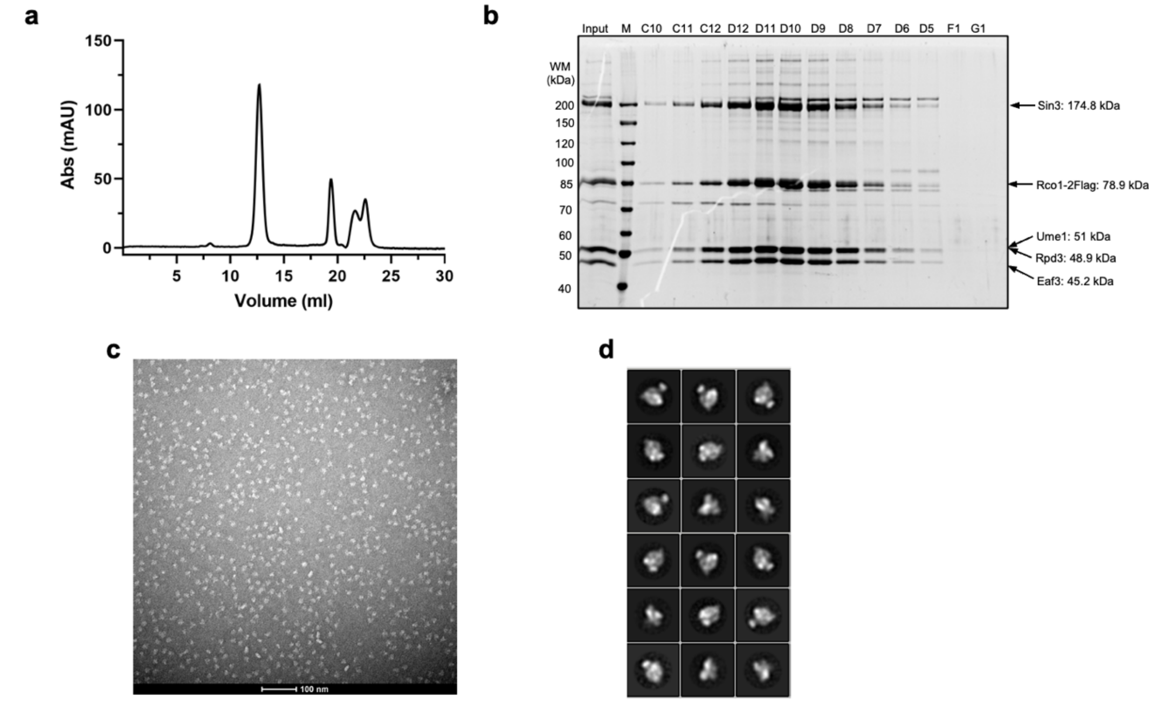

Fig. S1 Purification and preliminary EM analysis of the Rpd3S. a, Size-exclusion chromatogram of the endogenously purified Rpd3S from *S. cerevisiae,* using a Superose 6 10/300 GL gel filtration column and monitoring absorption at 280 nm. **b,** SDS-PAGE analysis of the purified Rpd3S (the first peak). **c,** A typical electron micrograph showing the appearance of the negative stained Rpd3S sample. **d,** A typical 2D class average of Rpd3S preserved under negative stain.

Supplementary information, Fig. S2

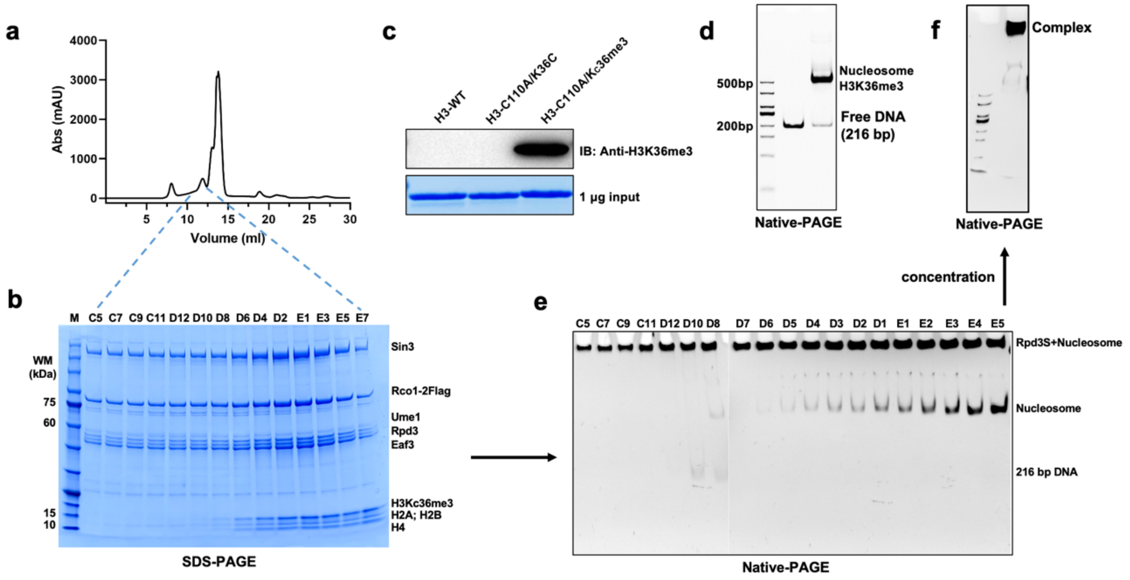

Fig. S2 Rpd3S-nucleosome complex assembly. a, Purification of the Rpd3S-nucleosome complex Superose 6 10/300 GL column. **b,** SDS-PAGE analysis of the Rpd3S-nucleosome complex peak (~12 ml). **c,** The identification of the H3Kc36me3-modified H3 was performed by Western blotting. **d,** The reconstituted mononucleosome with 216 bp DNA was assessed by Native-PAGE. **e,** The Rpd3S-nucleosome complex fractions were assessed by Native-PAGE and the fractions from C5 to E5 were selected for cryo-EM analysis. **f,** The concentrated Rpd3S-nucleosome complex was assessed by Native-PAGE before cryo-EM grids preparation.

Supplementary information, Fig. S3

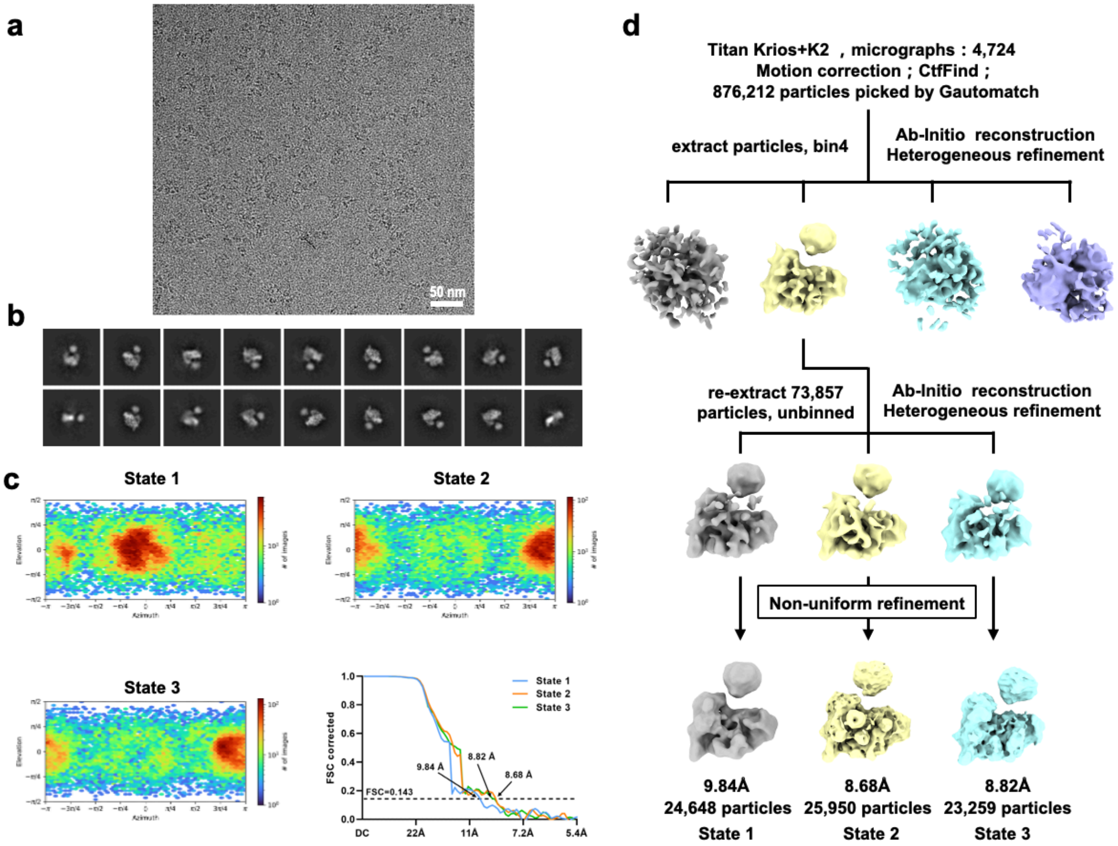

Fig. S3 Cryo-EM data processing of *S. cerevisiae* Rpd3S. a, A typical micrograph of the Rpd3S preserved in vitrified ice. Scale bar, 50 nm. **b,** Typical 2D class averages obtained after reference-free alignment and classification of images of Rpd3S particles. **c,** Heat maps showing particle orientation distribution and Gold-standard FSC curves of the reconstructed Rpd3S three conformations: state 1 map 9.84 Å, state 2 map 8.86 Å and state 3 map 8.82 Å (FSC = 0.143). **d,** Flow chart of cryo-EM data processing.

Supplementary information, Fig. S4

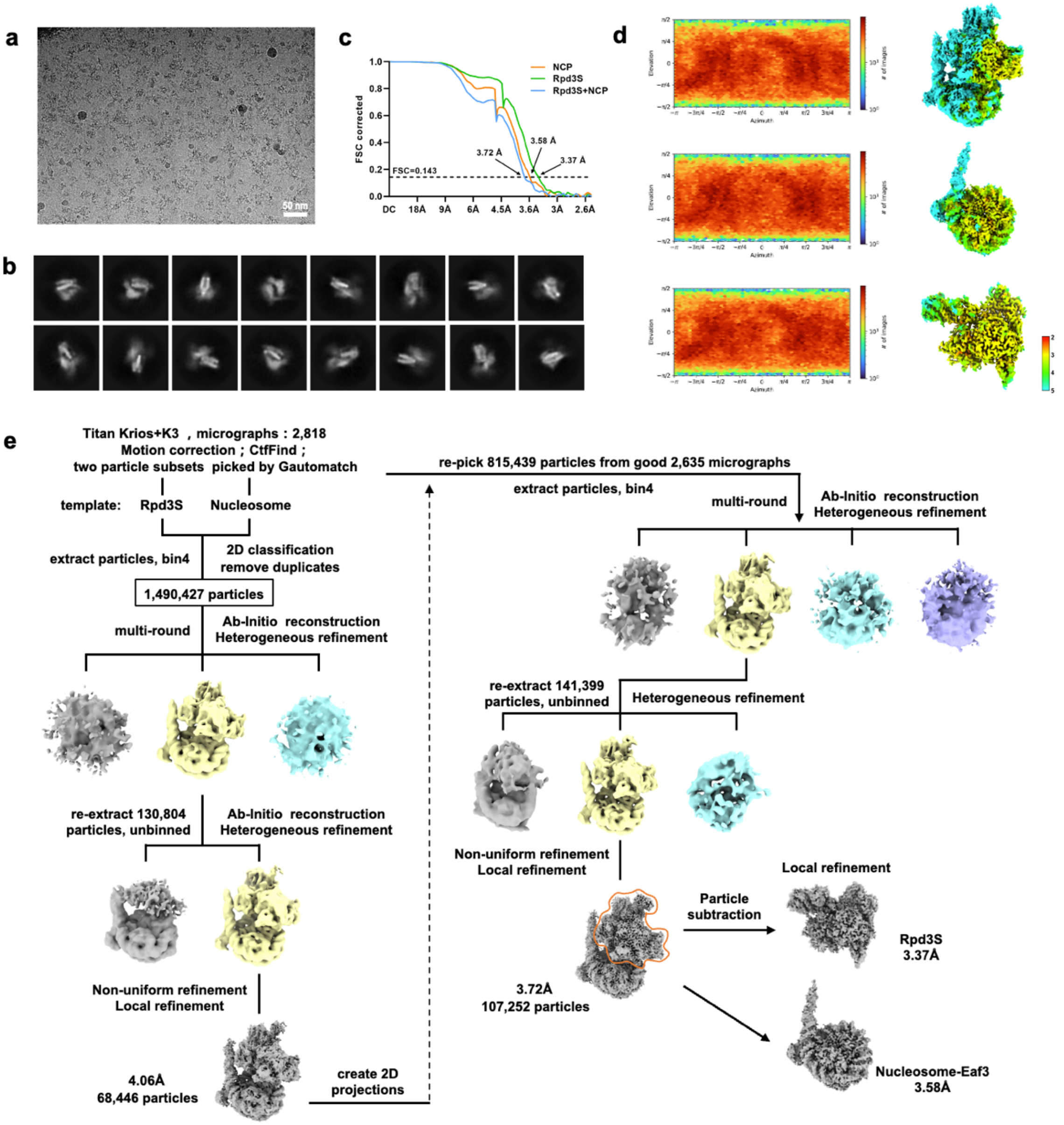

Fig. S4 Rpd3S-nucleosome complex cryo-EM reconstruction. a, Representative micrograph of the dataset used to determine the Rpd3S-nucleosome complex structure. Scale bar, 50 nm. **b,** A typical reference-free 2D classes averages of Rpd3S-nucleosome. **c,** FSC curve for the cryo-EM density map according to the gold-standard criterion. The final resolution of Rpd3S-nucleosome is 3.72 Å, Rpd3S core 3.37 Å, and nucleosome-Eaf3 3.58 Å. **d,** Angular distributions of particles used in the final 3D reconstruction (left) and the local resolution of the corresponding cryo-EM map (right), Rpd3S-nucleosome (top), nucleosome-Eaf3 (middle), Rpd3S core (bottom). **e,** Image processing scheme. Particles were picked using different templates to achieve higher map resolution, also performed particle subtraction and local refinement.

Supplementary information, Fig. S5

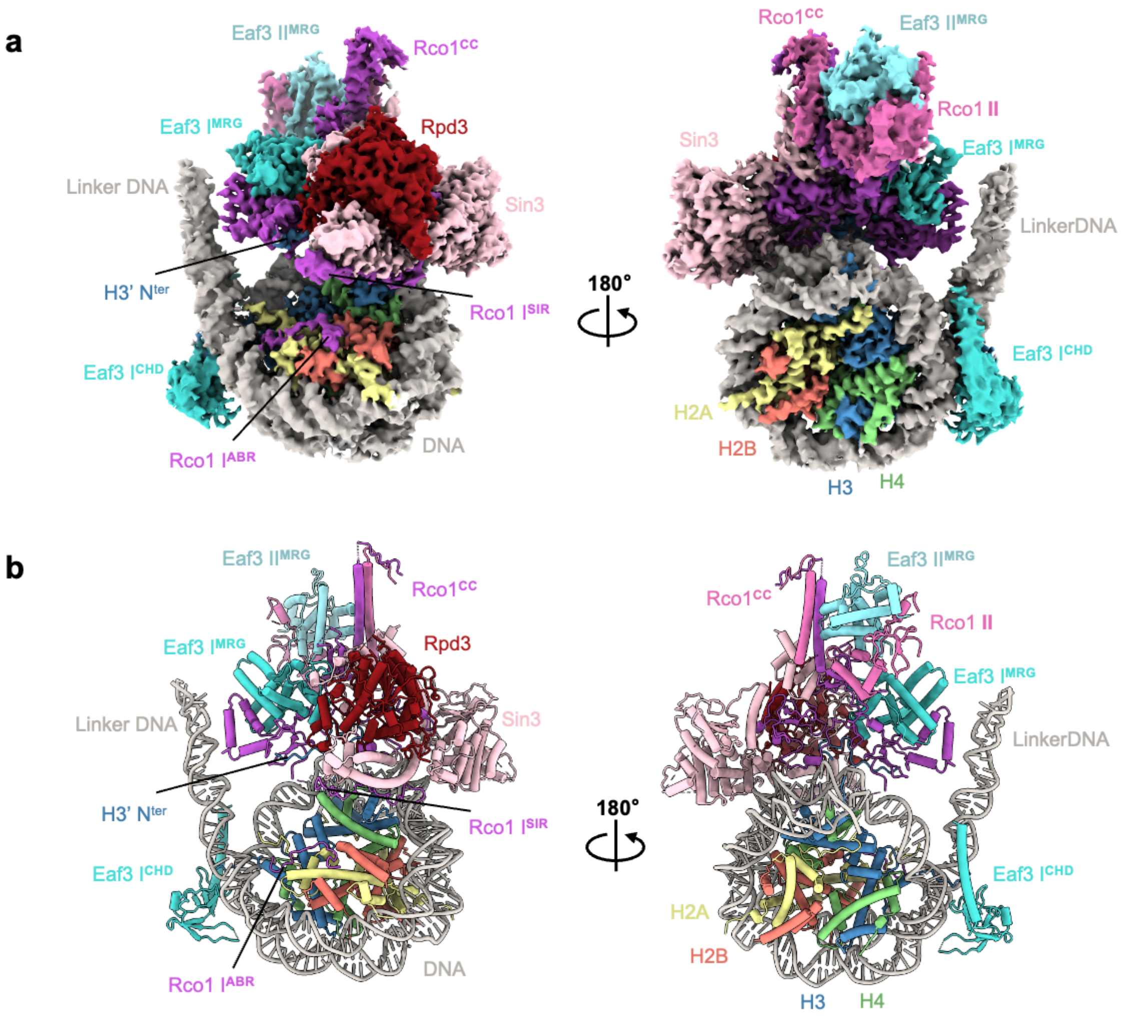

Fig. S5 Cryo-EM structure of the Rpd3S-nucleosome complex. a, Front (left) and back (right) views of the cryo-EM composite map (see Methods) of the Rpd3S-nucleosome complex. Mononucleosomes containing methyl-lysine analogs (H3K36me3) with 70bp DNA link at one end are used in the assembly. **b,** Corresponding views of the structural model of the Rpd3S-nucleosome complex.

Supplementary information, Fig. S6

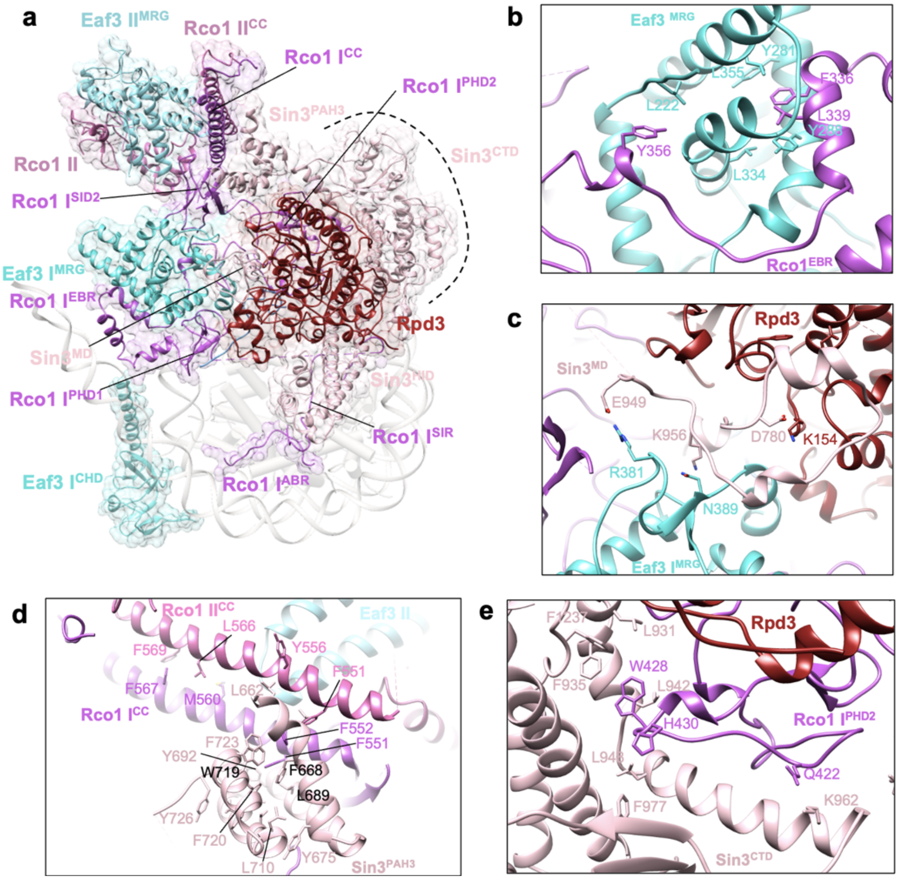

Fig. S6 Structural organization of Rpd3S. a, Overall architecture of Rpd3S highlighted in transparent surface representation within the Rpd3S-nucleosome complex. **b-e,** Detailed interactions in the Rco1 I^{EBR} - Eaf3 I^{MRG} (**b**), the $Sin3^{MD}$ - Rpd3 - Eaf3 I^{MRG} (**c**), the $Sin3^{PAH3}$ –$Rco1^{CC}$ (**d**) and the $Sin3^{CTD}$-Rco1 I^{PHD2} (**e**) interfaces.

Supplementary information, Fig. S7

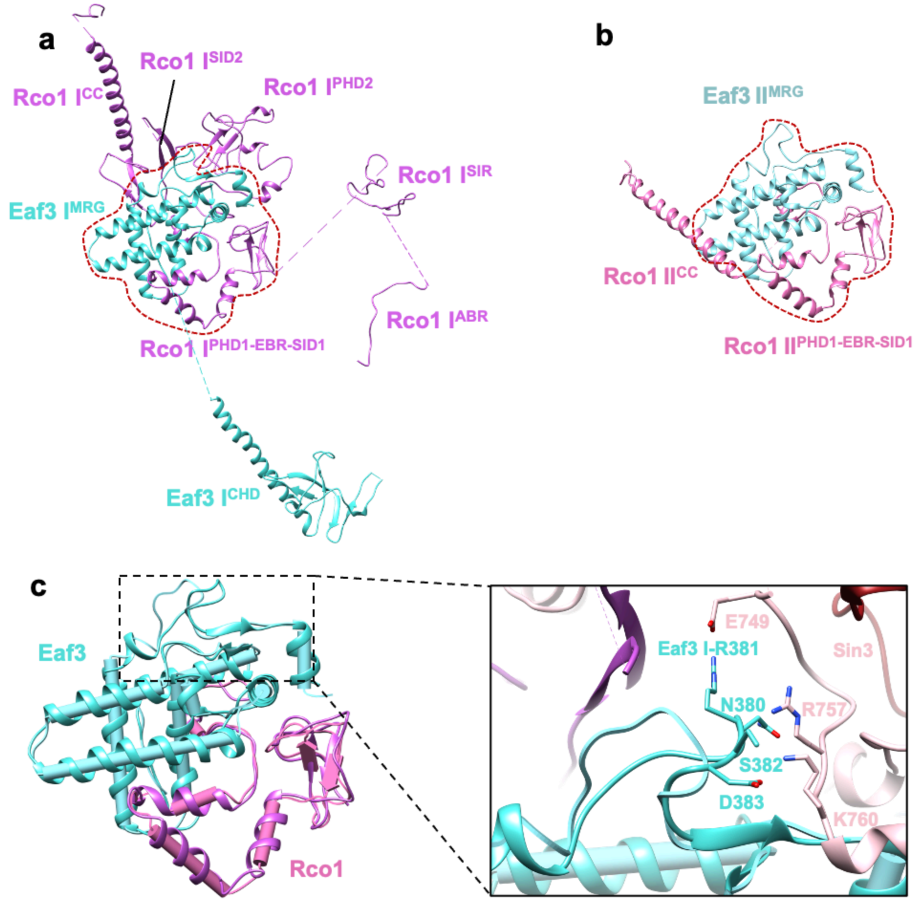

Fig. S7 The comparison between Eaf3-Rco1 two copies in Rpd3S-nucleosome complex. a-b, Eaf3 MRG domain and Rco1 PHD-EBR-SID1 domain of copy 1 are highlighted by the red dotted line, the same view of Eaf3-Rco1 copy 2 shown in **b**. **c,** Eaf3-Rco1 two copies are merged (left) and the close-up view of the significant difference in the loop of Eaf3 (375-383 residues) is shown (right).

Supplementary information, Fig. S8

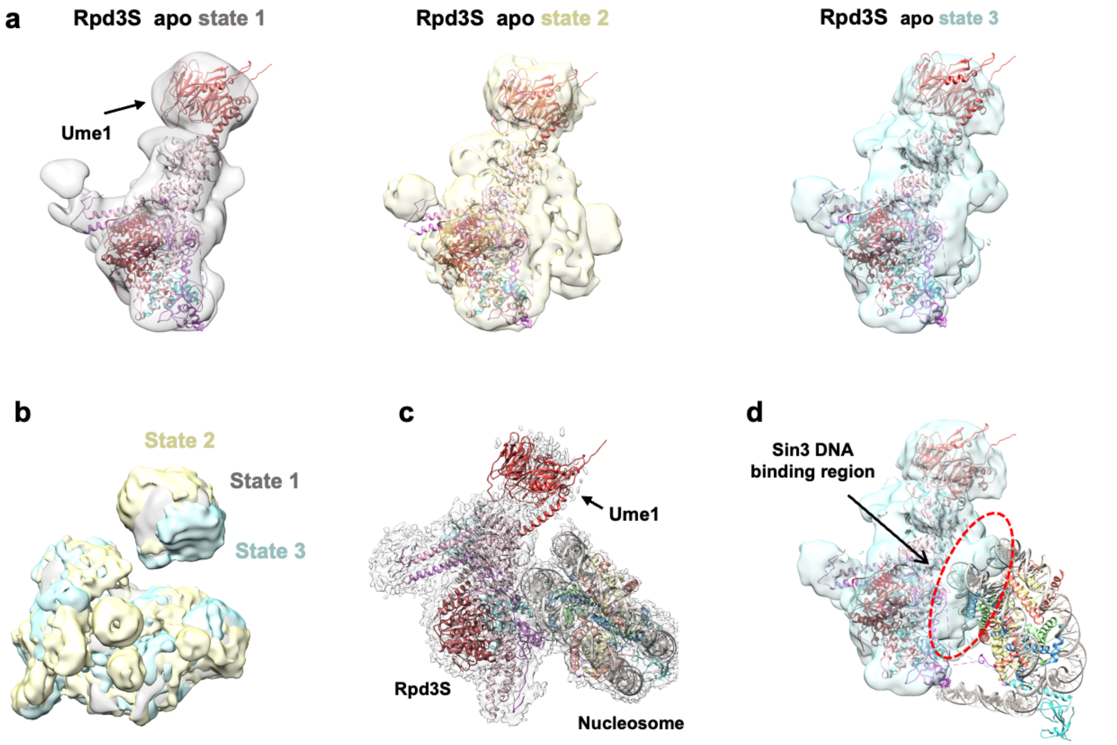

Fig. S8 The potential location of Ume1 in Rpd3S holoenzyme. a, Ume1 structure from AlphaFold protein structure database is denoted and fitted into the Rpd3S apo states maps. The low resolution EM map of the apo Rpd3S can better illustrate the localization and conformational dynamic of Ume1. **b,** The comparison between the Rpd3S apo states (shown in different colors). **c,** Ume1 fitted in the disordered region of Rpd3S-nucleosome complex. **d,** Sin3 DNA binding region is occupied by additional density (highlighted by the red dotted line) in Rpd3S apo state.

Supplementary information, Fig. S9

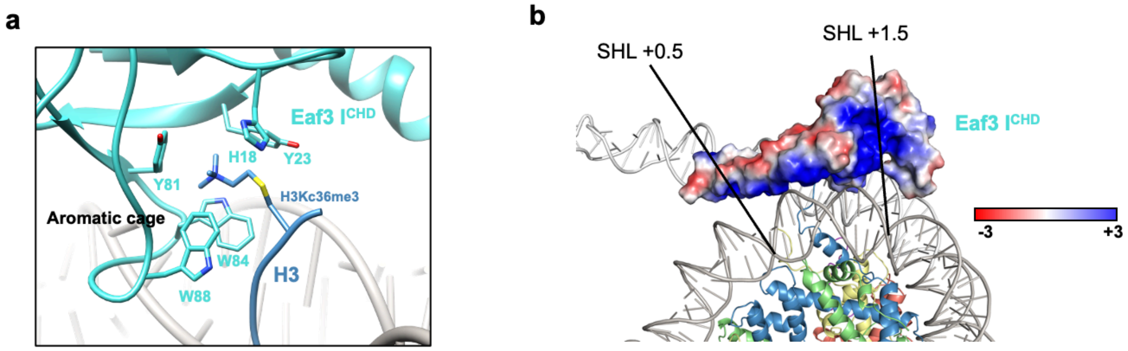

Fig. S9 Binding of Eaf3 CHD domain to H3K36me3 and nucleosome DNA. a, The close-up view of the interactions between the Eaf3-CHD aromatic cage and the H3Kc36me3 modified nucleosome. The residues of the aromatic cage and aromatic cage are shown as sticks. **b,** Details of DNA interactions. The positively charged surface is involved in DNA interaction. SHLs are denoted. Electrostatic surface of the Eaf3 CHD domain is calculated using the APBS tool.

Supplementary information, Fig. S10

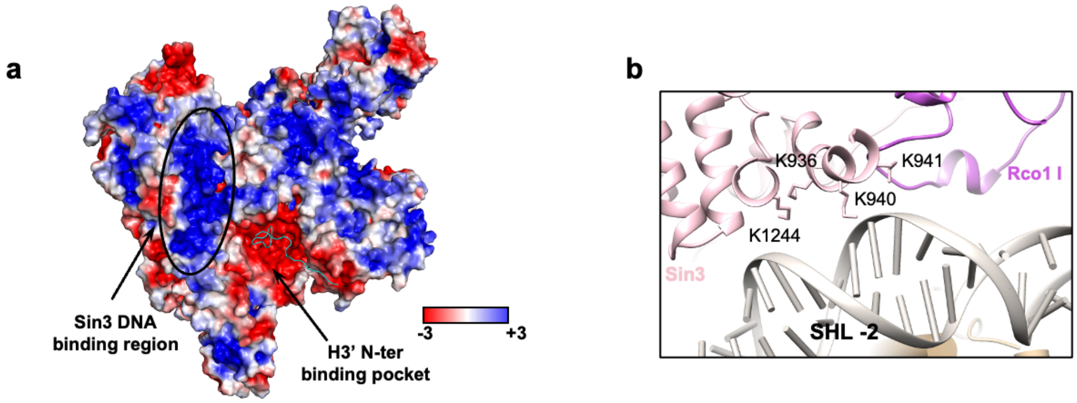

Fig. S10 Interface of Sin3 with nucleosome DNA. a, Electrostatic surface of the Rpd3S core complex calculated using the APBS tool in a range of -3 kT e^{-1} to +3 kT e^{-1}. The positively charged surface is consistent with the DNA binding region of Sin3, and the catalytic substrate H3’ N-ter (shown in ribbon) is deposited in the negatively charged pocket. **b,** Close-up view showing the interface of Sin3 with nucleosome DNA at SHL -2.

Supplementary information, Fig. S11

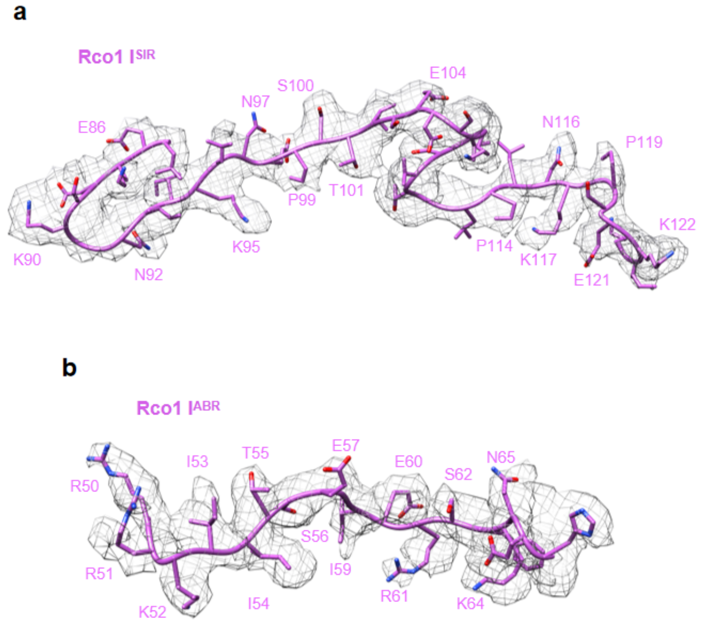

Fig. S11 Quality of the density maps and structural models of Rco1 SIR and ABR domain. a, The density map (grey mesh) of Rco1 SIR domain. The residues (85-124 residues) are shown as sticks. **b,** The density map of Rco1 ABR (50-67 residues) used for model building.

Supplementary information, Fig. S12

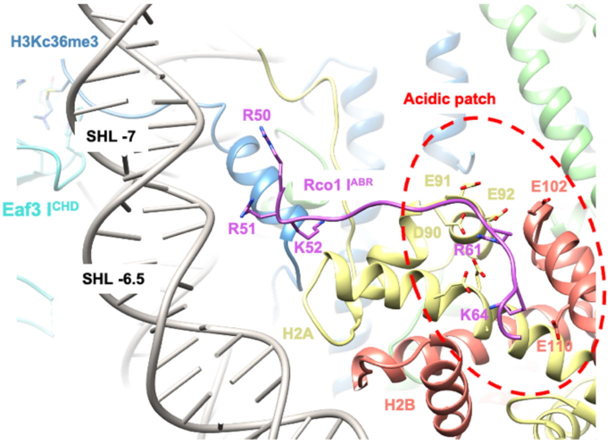

Fig. S12 The close-up view of the interactions between Rco1 ABR and nucleosome.
In addition to the acidic patch recognition (dotted red circle), the ABR domain could bind the nucleosome DNA at SHL -6.5. The residues are shown as sticks.

Supplementary information, Fig. S13

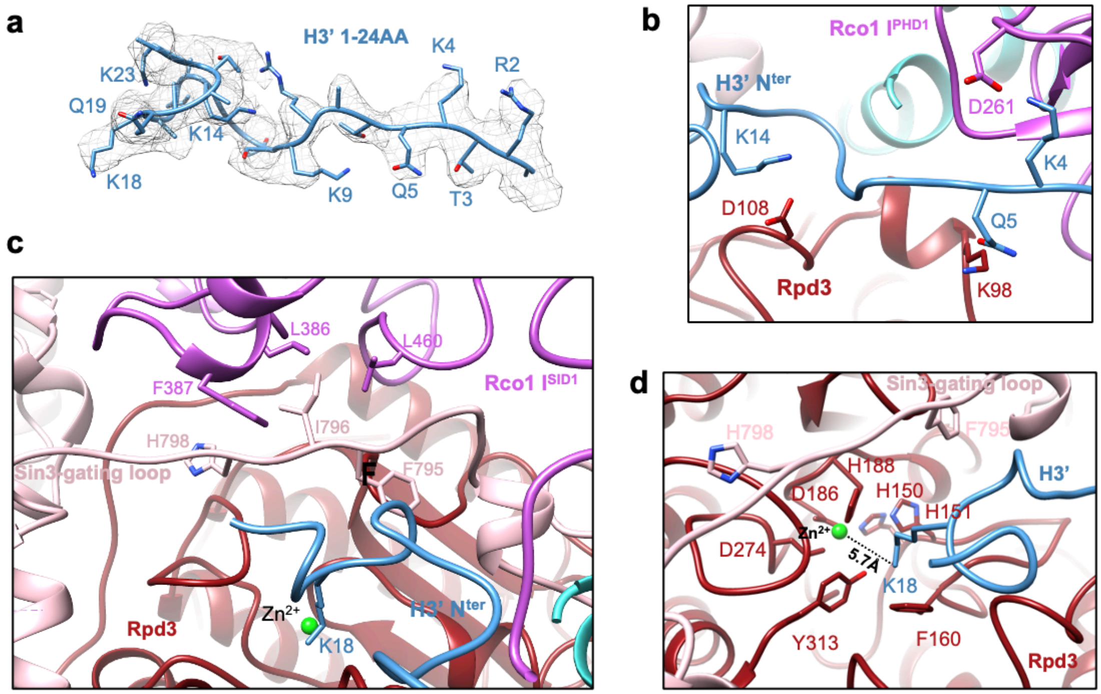

Fig. S13 The close-up view of the histone H3 substrate. a, Structure of H3 tail (1-24 aa) penetrating into the active center of Rpd3S are shown as transparent EM density map fitted with the corresponding model. **b,** Close-up view of H3 tail depicting how the H3K4 and H3Q5 are gripped by Rco1 I and Rpd3, respectively. **c,** The side chain of H3K18 deposited into the Rpd3 HDAC active core is shown, and the side chain of residues involved in the interface between Rco1-SID1 and Sin3-gating loop was denoted. **d,** Close view showing the ϵ-amino group of H3K18 residue in the active site is poised for catalysis. Rpd3 residues involved in interactions with H3 tail are depicted. Zn ion is highlighted in green. Sin3 gating loop doesn't interact with the H3 tail substrate.

Supplementary information, Fig. S14

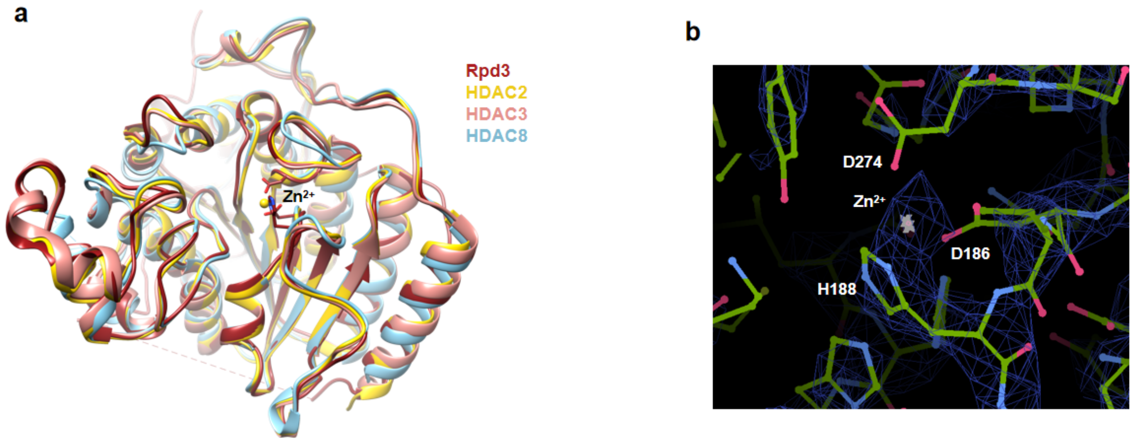

Fig. S14 Structural comparison between Rpd3 and HDACs. a, The comparison between Rpd3 and other known Class I HDAC structures. PDB codes of the structures used are: 6XEC (HDAC2), 4A69 (HDAC3) and 1W22 (HDAC8). **b,** Based on the comparison of homologous structures, the electron density map corresponding to zinc ion in the catalytic center can be easily determined in coot.

Supplementary information, Fig. S15

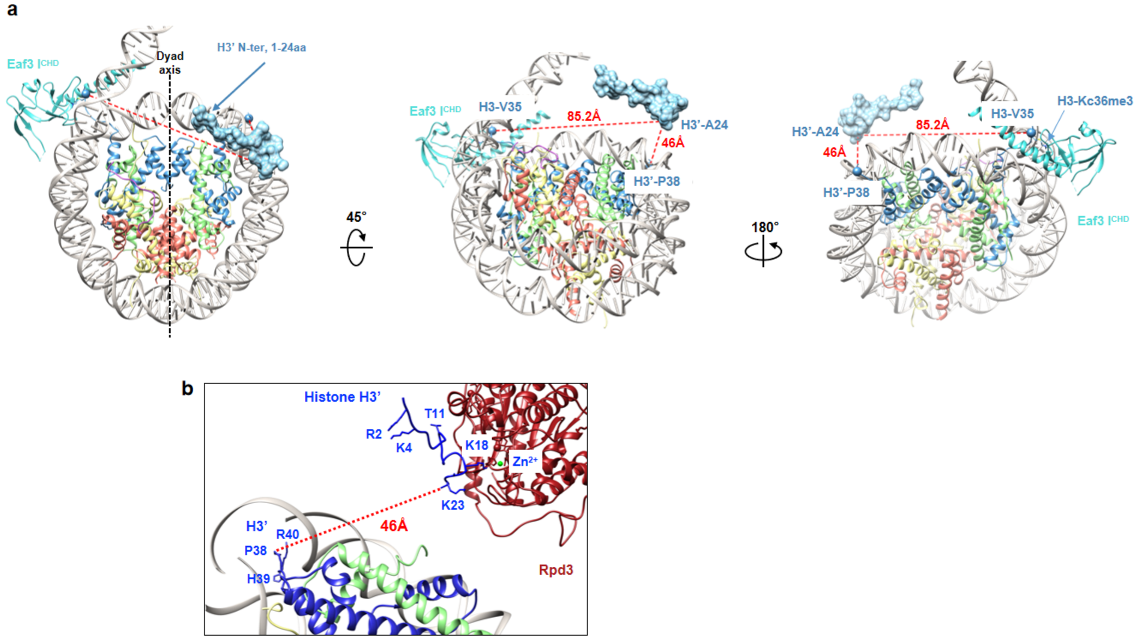

Fig. S15 The histone H3 tail deposited in the catalytic pocket is restrained in distance and different from the H3 bound by Eaf3 CHD. a, The histone H3' N-ter (1-24 residues) in catalytic site are shown as surface. The distances of the H3' A24 residue to the two H3 tails are shown in red with different views. **b,** The 46Å distance between H3'A24 and H3'P38 could allow 13 amino acid residues (25-37 aa) to adopt a straightened conformation.

Supplementary information, Fig. S16

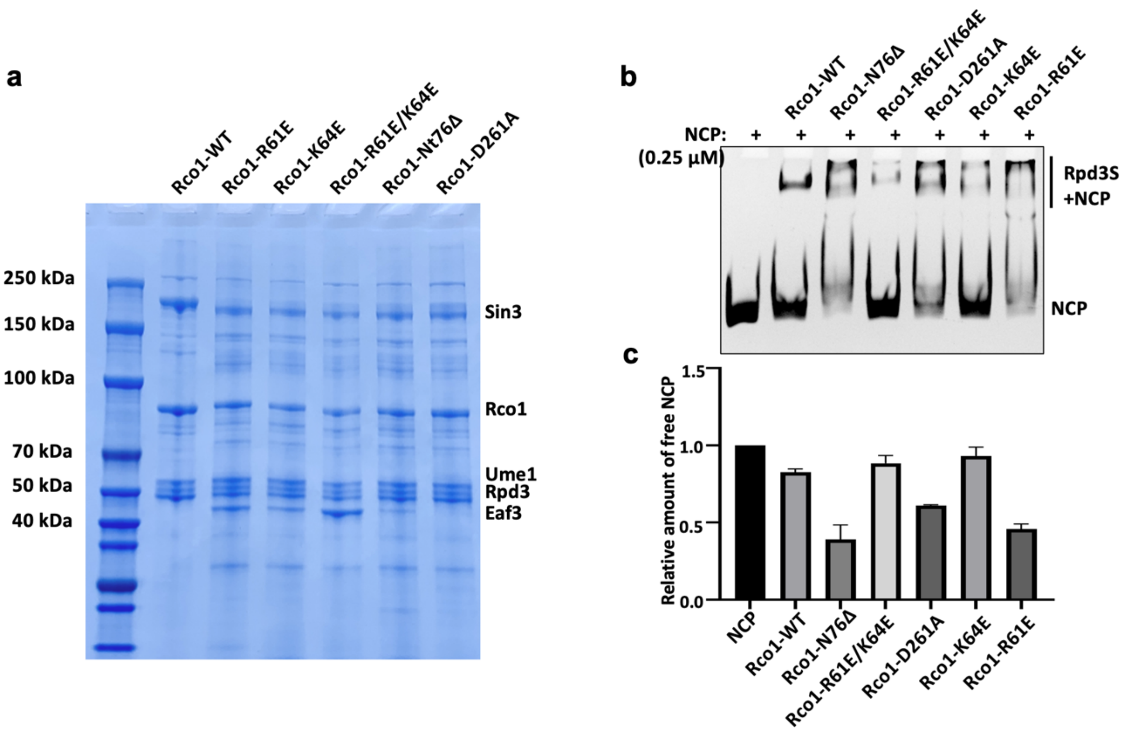

Fig. S16 Functional analysis of wild-type Rco1 and its mutants. a, Preparation of wild-type Rpd3S and its mutants from *S.cerevisiae*. SDS-PAGE gels were shown with Coomassie stain. All the endogenous purified Rpd3S were concentrated for the nucleosome binding and HDAC activity assay. **b,** EMSA analysis of the nucleosome binding activity of wild-type Rco1 and its mutants. 0.25 μM H3K36me3-modified nucleosome and equal amounts of Rpd3S complex were used. Rco1-N76Δ, Rco1 N-terminal 1-76aa deletion. **c,** The relative ratio of unbound nucleosome of wild-type Rco1 and its mutants. The ratio of unbound nucleosome of the experimental group to that of the control group was quantified on the same gel.

Supplementary information, Fig. S17

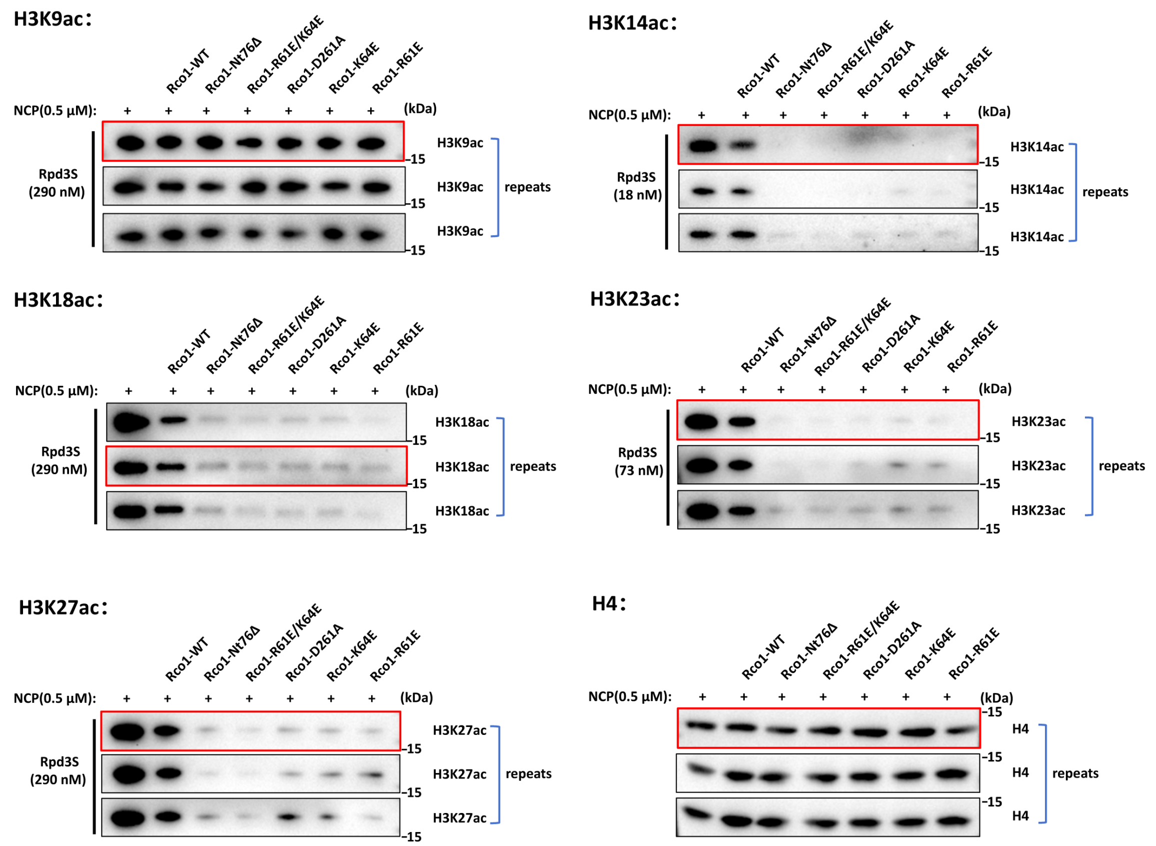

Fig. S17 Three independent *in vitro* HDAC assays of Rpd3S on nucleosome substrate. Western blot analysis of HDAC activity of WT Rpd3S and its mutants on H3K36me3 and H3/H4 hyperacetylated nucleosome. The concentration of nucleosome and the respective Rpd3S is indicated on the left.

Supplementary information, Fig. S18

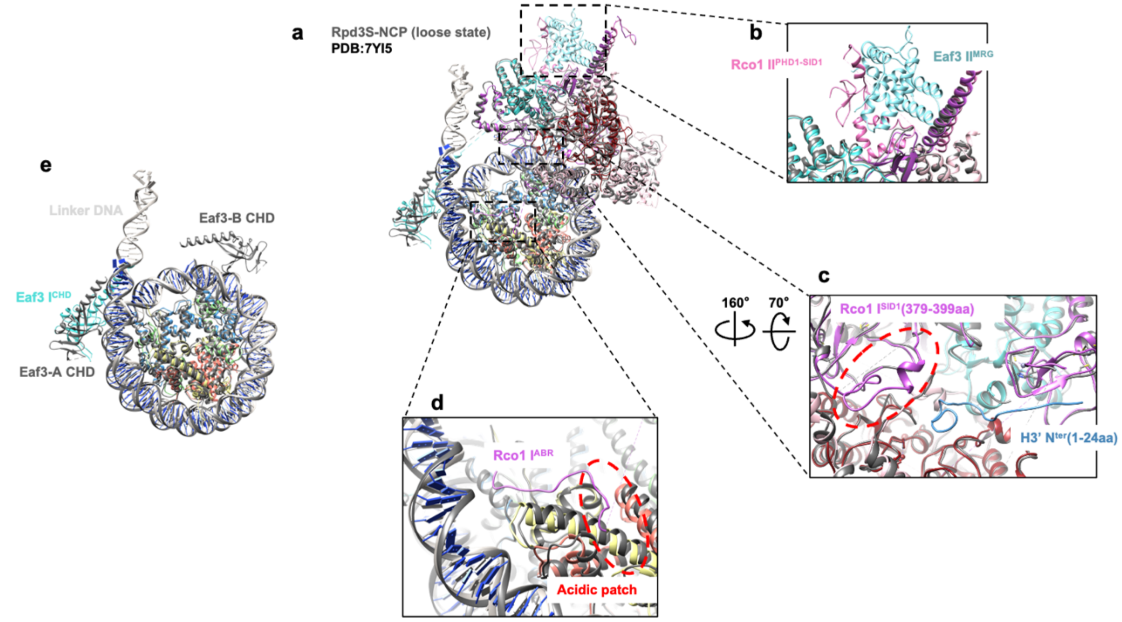

Fig. S18 Structural comparison of Rpd3S-NCP between the structures in this study and reported by Guan et al. The reported loose state Rpd3S-NCP structure (PDB:7YI5, showed as gray) is compared with our reconstruction (colored as in Fig. 1a) (**a**). Our model provides additional structural information: Eaf3-Rco1 copy II containing Eaf3 MRG and Rco1 PHD1-SID1 (**b**), the intact H3 tail (1-24 residues), Rco1 I^{SID1} containing 379-399 residues (**c**) and Rco1 I^{ABR} (50-67 residues) binding nucleosome acidic patch (**d**). **e,** Only one copy of Eaf3 bound to linker DNA is observed in our structure, and no other copy is observed.

Supplementary information, Table S1 Cryo-EM data collection, refinement and validation statistics.

	NCP-Eaf3 (PDB 8IHM) (EMDB-35449)	Rpd3S core (PDB 8IHN) (EMDB-35450)	Rpd3S-NCP (PDB 8IHT) (EMDB-35455)	Rpd3S apo state 1 (EMDB-35456)	Rpd3S apo state 2 (EMDB-35457)	Rpd3S apo state 3 (EMDB-35458)
**Data collection and processing**						
Microscope	Krios G3	Krios G3	Krios G3	Krios G3	Krios G3	Krios G3
Camera	K3	K3	K3	K2	K2	K2
Voltage (kV)	300	300	300	300	300	300
Magnification	81,000	81,000	81,000	18,000	18,000	18,000
Electron exposure ($e^-/Å^2$)	53	53	53	59	59	59
Pixel size (Å)	1.07	1.07	1.07	1.35	1.35	1.35
Frames (no.)	32	32	32	36	36	36
Defocus range (μm)	1.8-2.8	1.8-2.8	1.8-2.8	2.5-3.5	2.5-3.5	2.5-3.5
Symmetry imposed	C1	C1	C1	C1	C1	C1
Micrographs (no.)	2,818	2,818	2,818	4,724	4,724	4,724
Initial particle images (no.)	815,439	815,439	815,439	876,212	876,212	876,212
Final particle images (no.)	107,252	107,252	107,252	24,648	25,950	23,259
Map resolution (Å)	3.58	3.37	3.72	9.84	8.68	8.82
FSC threshold	0.143	0.143	0.143	0.143	0.143	0.143
**Model building**						
Software	Coot	Coot	Coot			
**Refinement**						
Software	Phenix	Phenix	Phenix			
Model-map scores						
CC (mask)	0.83	0.81	0.72			
CC (box)	0.84	0.76	0.80			
CC (peaks)	0.78	0.71	0.69			
CC (volume)	0.82	0.80	0.72			
**Validation**						
MolProbity score	1.82	1.72	1.67			
Clash score	10.55	7.39	6.54			
Rotamer outliers (%)	0.00	0.32	0.23			
Cβ outliers (%)	0.00	0.00	0.00			
CaBLAM outliers (%)	0.97	2.66	1.71			
**R.m.s deviations**						
Bonds length (Å)	0.007	0.005	0.006			
Bonds Angle (°)	0.705	0.724	0.917			
**Ramachandran plot**						
Favored (%)	95.93	95.47	95.62			
Allowed (%)	4.07	4.47	4.34			
Outlier (%)	0.00	0.05	0.04			
